# Supplementary material for: Adverse Pregnancy Outcomes and Maternal Chronic Diseases in the Future: A Cross-Sectional Study Using KoGES-HEXA Data
Source: J Clin Med. 2022 Mar 7;11(5):1457. doi: 10.3390/jcm11051457 (PMC8911450; doi:10.3390/jcm11051457)
Supplement: Supplementary file 1 [file jcm-11-01457-s001.zip › jcm-1574879-supplementary.pdf]

Supplementary Materials

Table S1. Logistic regression analysis for APOs for predicting chronic disease.

	Hypertension	Diabetes mellitus	Hyperlipidemia	Transient ischemia	Stroke	Angina pectoris
	OR (95% CI)	OR (95% CI)	OR (95% CI)	OR (95% CI)	OR (95% CI)	OR (95% CI)
Composite APOs	0.979 (0.906, 1.058)	1.184 (1.042, 1.344)	1.177 (1.048, 1.322)	0.692 (0.294, 1.631)	1.225 (0.942, 1.593)	1.127 (0.913, 1.390)
Preeclampsia	1.380 (1.219, 1.562)	1.055 (0.839, 1.326)	1.249 (1.027, 1.518)	1.312 (0.407, 4.229)	1.311 (0.848, 2.025)	1.336 (0.954, 1.872)
GDM	0.896 (0.635, 1.265)	3.780 (2.680, 5.333)	1.291 (0.806, 2.067)	-	0.739 (0.183, 2.983)	0.442 (0.110, 1.780)
Stillbirth	1.378 (1.023, 1.857)	1.272 (0.764, 2.117)	0.959 (0.568, 1.620)	-	2.198 (0.971, 4.973)	1.081 (0.444, 2.632)
Macrosomia	0.778 (0.694, 0.871)	1.371 (1.165, 1.614)	1.151 (0.982, 1.347)	0.236 (0.033, 1.714)	1.188 (0.832, 1.697)	1.042 (0.777, 1.396)
LBW	0.963 (0.837, 1.107)	0.734 (0.559, 0.964)	1.078 (0.872, 1.332)	1.382 (0.429, 4.458)	1.180 (0.741, 1.880)	1.043 (0.711, 1.530)
Preeclampsia only	1.403 (1.236, 1.592)	1.031 (0.813, 1.309)	1.301 (1.068, 1.585)	1.373 (0.426, 4.428)	1.381 (0.894, 2.134)	1.364 (0.969, 1.919)
GDM only	0.889 (0.584, 1.355)	4.796 (3.258, 7.059)	1.768 (1.067, 2.930)		1.109 (0.274, 4.491)	0.330 (0.046, 2.355)
Preeclampsia + GDM	0.961 (0.528, 1.749)	1.900 (0.871, 4.143)	0.408 (0.100, 1.662)			0.707 (0.098, 5.095)

Abbreviations: aOR, adjusted odds ratio; CI, confidence interval; APOs, adverse pregnancy outcome; GDM, gestational diabetes; LBW, low birth weight.

Table S2. Logistic regression analysis for APOs for predicting metabolic syndrome and its components.

	**Metabolic syndrome**	**Abdominal obesity**	**Elevated BP**	**Elevated FBS**	**Low HCL-C**	**High TG**
	**OR (95% CI)**	**OR (95% CI)**	**OR (95% CI)**	**OR (95% CI)**	**OR (95% CI)**	**OR (95% CI)**
Composite APOs	0.957 (0.888, 1.031)	1.036 (0.970, 1.108)	0.964 (0.907, 1.025)	1.108 (1.034, 1.187)	0.951 (0.890, 1.015)	0.958 (0.891, 1.031)
Preeclampsia	1.073 (0.944, 1.219)	1.123 (1.002, 1.259)	1.319 (1.188, 1.465)	1.055 (0.934, 1.191)	0.926 (0.825, 1.039)	0.986 (0.868, 1.119)
GDM	1.136 (0.834, 1.548)	0.945 (0.705, 1.267)	0.934 (0.717, 1.216)	1.972 (1.513, 2.572)	0.982 (0.741, 1.302)	0.976 (0.713, 1.336)
Stillbirth	1.335 (0.998, 1.786)	1.269 (0.969, 1.663)	1.484 (1.154, 1.910)	1.215 (0.915, 1.613)	1.071 (0.816, 1.406)	1.315 (0.989, 1.749)
Macrosomia	0.946 (0.853, 1.048)	1.193 (1.092, 1.304)	0.804 (0.738, 0.875)	1.222 (1.114, 1.340)	0.977 (0.894, 1.069)	0.919 (0.831, 1.016)
LBW	0.810 (0.703, 0.933)	0.728 (0.640, 0.828)	0.941 (0.844, 1.050)	0.845 (0.741, 0.963)	0.894 (0.794, 1.008)	0.980 (0.860, 1.116)
Preeclampsia only	1.068 (0.937, 1.218)	1.114 (0.991, 1.253)	1.337 (1.201, 1.488)	1.041 (0.918, 1.179)	0.940 (0.835, 1.058)	0.977 (0.858, 1.113)
GDM only	1.123 (0.771, 1.636)	0.815 (0.564, 1.178)	0.915 (0.664, 1.261)	2.259 (1.646, 3.101)	1.135 (0.814, 1.581)	0.902 (0.612, 1.330)
Preeclampsia + GDM	1.176 (0.684, 2.021)	1.275 (0.784, 2.072)	1.016 (0.640, 1.614)	1.459 (0.892, 2.384)	0.691 (0.402, 1.187)	1.139 (0.670, 1.937)

Abbreviations: BP, blood pressure; FBS, fasting blood sugar; HDL-C, high-density lipoprotein cholesterol; TG, triglyceride; aOR, adjusted odds ratio; CI, confidence interval; APO, adverse pregnancy outcome; GDM, gestational diabetes; LBW, low birth weight.
